# Successful Endovascular Repair of an Iatrogenic Perforation of the Superficial Femoral Artery Using Self-Expanding Nitinol Supera Stents in a Patient with Acute Thromboembolic Limb Ischemia

**DOI:** 10.1155/2016/7376457

**Published:** 2016-04-24

**Authors:** Tom Eisele, Benedikt M. Muenz, Grigorios Korosoglou

**Affiliations:** Department of Cardiology & Vascular Medicine, GRN Hospital Weinheim, 69469 Weinheim, Germany

## Abstract

The treatment of acute thromboembolic limb ischemia includes well-established surgical thrombectomy procedures and, in recent times, also percutaneous rotational thrombectomy using Straub Rotarex® system. This modality not only enables efficient treatment of such thrombotic occlusion but also in rare cases may imply the risk of perforation of the occluded artery. Herein, we report the case of a perforation of the superficial femoral artery (SFA) in an elderly female patient with thromboembolic limb ischemia. The perforation was successfully treated by implantation of self-expanding nitinol Supera stents and without the need for implantation of a stent graft.

## 1. Introduction

Peripheral artery disease (PAD) exhibits increasing morbidity and mortality during the last few decades [1] and is estimated to affect over 200 million people worldwide, including up to 30% of elderly primary care individuals [2, 3]. Patients with symptomatic PAD exhibit a life expectancy of 80% within 5 years of follow-up [4, 5], whereas patients with critical limb ischemia (CLI) show significant poorer outcomes with amputation and death rates of 15–20% and 25%, respectively, within the first year after diagnosis [6].

Especially patients with acute thromboembolic limb ischemia are in high risk of major amputation and death due to sepsis and multiorgan dysfunction. Such patients are usually older than 75 years and show further comorbidities, including atrial fibrillation and history of heart failure [7]. The treatment of this limb- and life-threatening condition includes well-established surgical thrombectomy procedures and, in recent times, also percutaneous rotational thrombectomy procedures using Straub Rotarex system, if required in combination with local thrombolysis [8]. This method enables quick and efficient treatment of peripheral arterial thromboembolic occlusion. Despite the low complication rate of rotational thrombectomy, perforations of the occluded artery have been reported previously. Herein, we report the case of a perforation of the superficial femoral artery (SFA) in an elderly female patient with thromboembolic limb ischemia, which was successfully treated by implantation of self-expanding nitinol Supera stents.

## 2. Case Presentation

**Figure 1:**
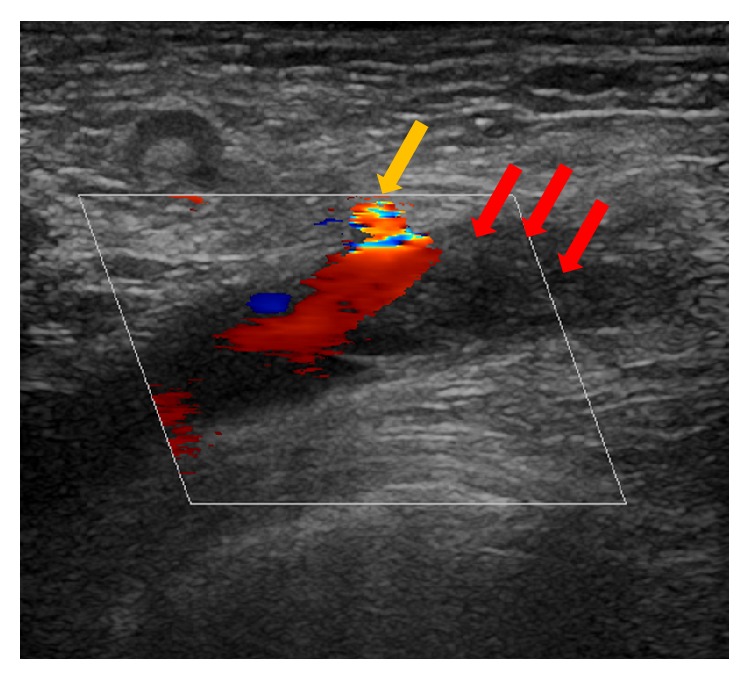
Thrombotic occlusion of the left common femoral artery (CFA). Absence of flow can be appreciated in the distal part of the vessel, associated with thrombus formation (red arrows). The origin of a collateral artery can be depicted proximally to the vessel occlusion (yellow arrow).

**Figure 2:**
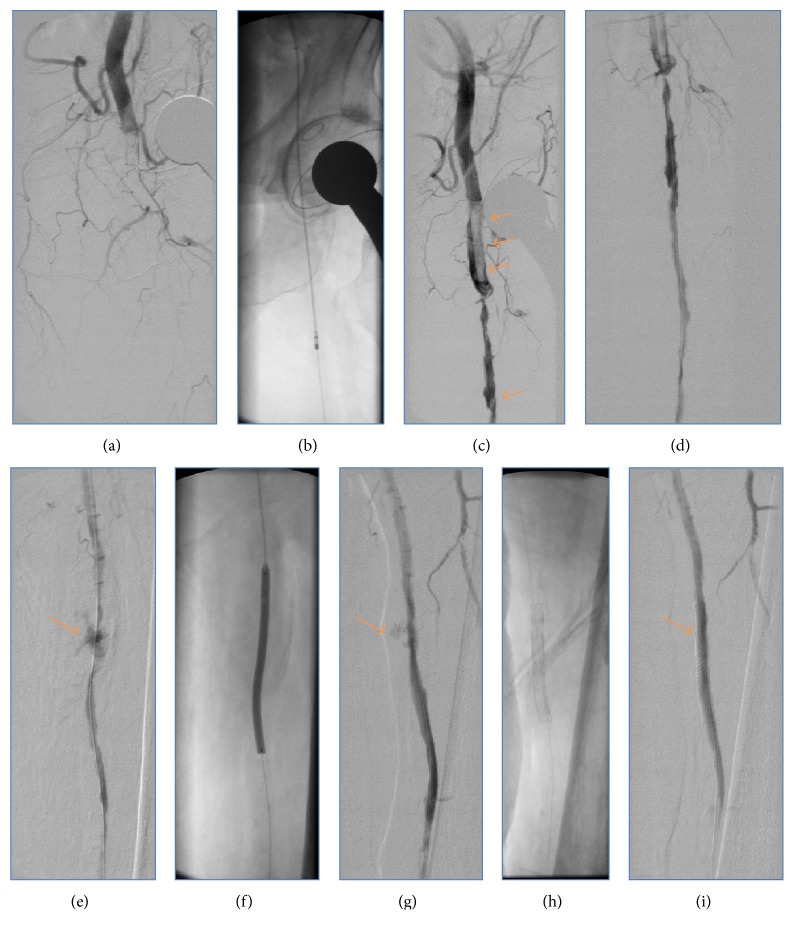
Occlusion of the CFA is noticed by DSA (a). Rotarex catheter thrombectomy reestablishes antegrade flow in the CFA and SFA (b), but thrombotic burden remains high as it can be appreciated by the orange arrows in (c). Mid and distal parts of the SFA with thrombus formations can also be appreciated in (d). After repeated Rotarex thrombectomy a perforation of the SFA is noticed, with persistent despite prolonged balloon inflation (orange arrows in (e)–(g)). Subsequently, 2 overlapping nitinol Supera stents are implanted (h), resulting in complete cessation of the bleeding complication (orange arrow in (i)).

**Figure 3:**
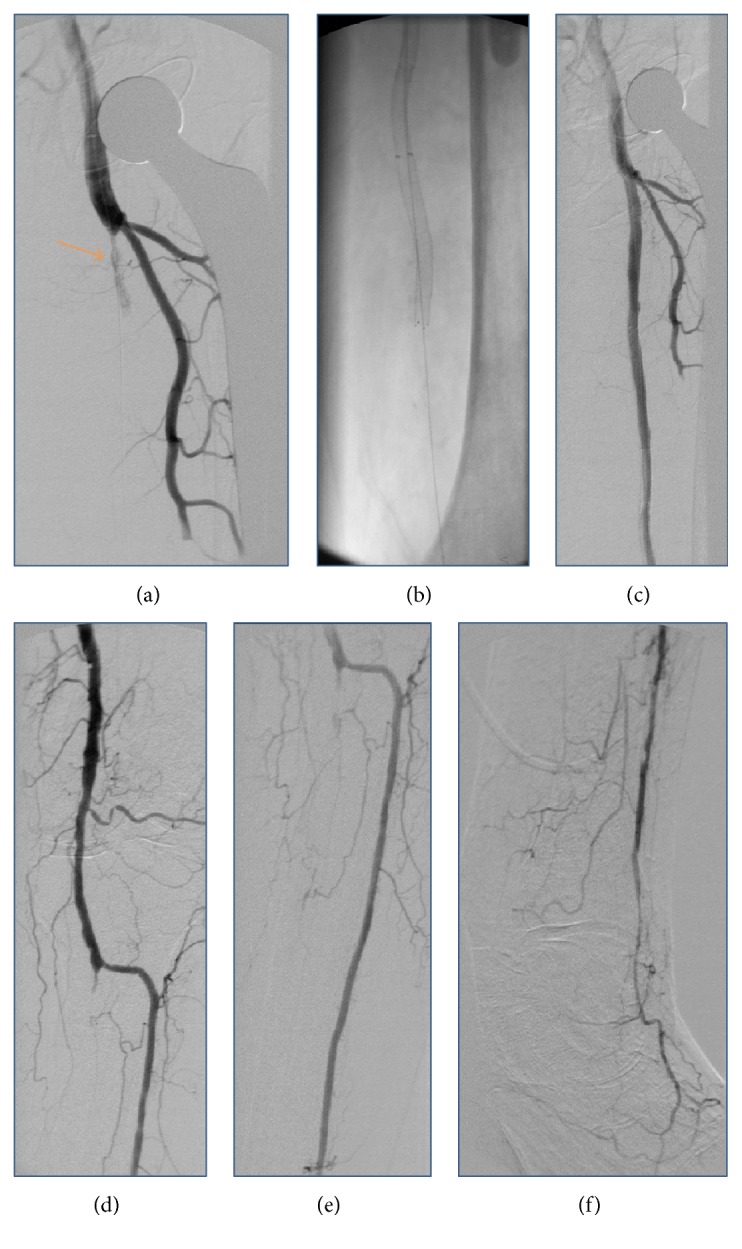
During the second angiographic session, DSA reveals not only complete resolution of the thrombus in the CFA (a) but also reocclusion of the SFA (orange arrow in (a)), requiring repeated Rotarex thrombectomy and implantation of further self-expanding nitinol stents (b). Subsequently, good angiographic flow can be achieved in the SFA and in the popliteal artery with a 1-vessel run-off of the anterior tibial artery ((c)–(f)).

**Figure 4:**
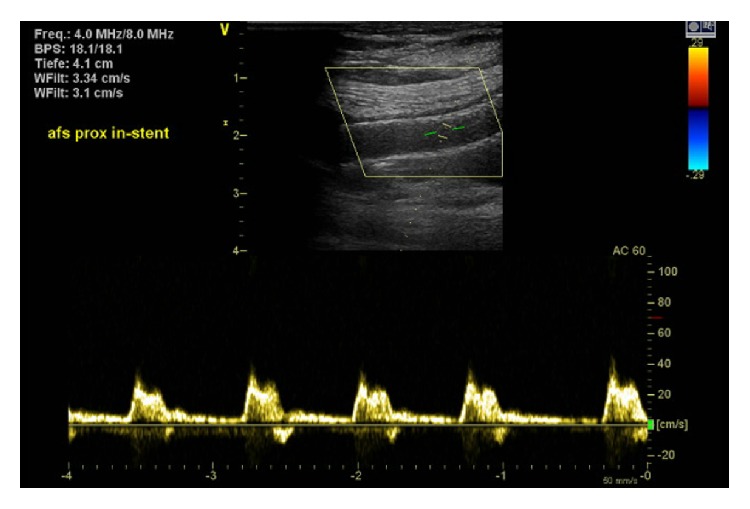
Triphasic flow in the SFA at 4 weeks of follow-up.

An 85-year-old female patient was referred to our department with new onset of severe pain, paleness, and pulselessness of her left leg since 12 hours. Duplex-sonography revealed thrombotic occlusion of the left common femoral artery (CFA) (Figure 1) and the patient was immediately scheduled for digital subtraction angiography (DSA). The patient had history of arterial hypertension and heart failure but no history of clinically evident PAD. DSA confirmed the thrombotic occlusion of the CFA (Figure 2(a)), and interventional treatment was initiated after injection of 500 mg aspirin and 5,000 I.U. of heparin. After insertion of an 0.018′′ guide wire, Rotarex catheter (ab medica GmbH, Düsseldorf, Germany) thrombectomy was performed in the CFA and in the proximal superficial femoral artery (SFA) (Figure 2(b)), resulting in antegrade flow in both SFA, but with high remaining thrombotic burden in the CFA (Figures 2(c) and 2(d)). After repeated Rotarex catheter thrombectomy a perforation of the SFA was noticed, which persisted despite prolonged inflation using a 5.0*∗*120 mm balloon (Figures 2(e)–2(g)). Due to persistent perforation, the implantation of 2 overlapping 5.0*∗*40 mm nitinol Supera stents (Abbott Vascular, Illinois, USA) was performed, which resulted in complete repair of the vascular injury of the SFA (Figures 2(h) and 2(i)). Local lysis was subsequently performed due to high remaining thrombotic burden of the CFA, using 10 mg bolus injection of Actilyse® (recombinant tissue plasminogen activator, Boehringer Ingelheim Pharma GmbH, Ingelheim am Rhein, Germany) and continuous infusion of 1 mg/h for further 16 hours. Repeated DSA was scheduled for the next day. In the mean time, pain symptoms of the patient improved, whereas she recovered from paleness of the lower limb except for the area of her distal foot. The next day, DSA revealed complete resolution of the thrombus in the CFA (Figure 3(a)). However, reocclusion of the SFA was unfortunately noticed (Figure 3(a), orange arrow), requiring repeated Rotarex catheter thrombectomy and implantation of 2 further self-expanding nitinol Innova*™* stents (7.0*∗*80 mm and 6.0*∗*120 mm, Boston Scientific, Ratingen, Germany) in the SFA (Figure 3(b)). Subsequently, good angiographic flow was shown in the SFA and in the popliteal artery with a 1-vessel run-off of the anterior tibial artery (Figures 3(c)–3(f)). After the second angiographic procedure pain symptoms and limb paleness and paresthesia were completely resolved. In addition, holter monitoring revealed the presence of atrial fibrillation and the patient was put on treatment with aspirin (100 mg daily), clopidogrel (75 mg daily), and 5 mg subcutaneous FXa-inhibitor fondaparinux for 4 weeks. Ambulatory Duplex-sonography at 4 weeks of follow-up demonstrated a good triphasic flow pattern in the SFA (Figure 4) and the patient was put on treatment with coumadin.

## 3. Discussion

To the best of our knowledge this is the first case reporting the placement of self-expanding nitinol Supera stent for the management of a perforation in a peripheral artery.

In the past decades, significant technical developments have occurred with endovascular therapy of acute thromboembolism, which offer several advantages over open surgical embolectomy techniques for the treatment of peripheral arterial thromboembolic occlusion [8]. Although the Straub Rotarex system is widely available and well established for the treatment of such thrombotic lesions, perforations of the occluded artery have been described previously and in our case. In such cases, usually stent grafts are employed. However, such stent grafts are expensive and are usually deliverable using ≥8F sheaths. This may require an additional arterial puncture under full anticoagulation or lead to additional time spent for changing the arterial sheath, which may aggravate bleeding complications. In our case, the SFA perforation could be successfully treated using self-expanding nitinol Supera stents, which are deliverable using a 6F sheath.

## 4. Conclusion

Herein we report the placement of a self-expanding nitinol Supera stent for the management of a perforation in a peripheral superficial femoral artery. Further novel options of complication management as described in our case may shift the treatment from surgical to even more endovascular treatment procedures in the future.
